# Phase II study of S-1 in patients with advanced biliary tract cancer

**DOI:** 10.1038/sj.bjc.6602208

**Published:** 2004-10-26

**Authors:** H Ueno, T Okusaka, M Ikeda, Y Takezako, C Morizane

**Affiliations:** 1 Hepatobiliary and Pancreatic Oncology Division, National Cancer Center Hospital, 5-1-1 Tsukiji, Chuo-ku, Tokyo 104-0045, Japan

**Keywords:** S-1, phase II study, biliary tract cancer, chemotherapy, pharmacokinetics

## Abstract

The aim of this study was to investigate the efficacy and safety of an oral fluoropyrimidine derivative, S-1, in patients with advanced biliary tract cancer. Patients with pathologically confirmed advanced biliary tract cancer, a measurable lesion, and no history of radiotherapy or chemotherapy were enrolled. S-1 was administered orally (40 mg m^−2^ b.i.d.) for 28 days, followed by a 14-day rest period. A pharmacokinetic study was performed on day 1 in the initial eight patients. In all, 19 consecutive eligible patients were enrolled in the study between July 2000 and January 2002. The site of the primary tumour was the gallbladder (*n*=16), the extrahepatic bile ducts (*n*=2), and the ampulla of Vater (*n*=1). A median of two courses of treatment (range, 1–12) was administered. Four patients achieved a partial response, giving an overall response rate of 21.1%. The median time-to-progression and median overall survival period were 3.7 and 8.3 months, respectively. Although grade 3 anorexia and fatigue occurred in two patients each (10.5%), no grade 4 toxicities were observed. The pharmacokinetic parameters after a single oral administration of S-1 were similar to those of patients with other cancers. S-1 exhibits definite antitumour activity and is well tolerated in patients with advanced biliary tract cancer.

The incidence of biliary tract cancer has been steadily increasing in Japan over the past several decades (Okusaka, 2002). Currently, biliary tract cancer is the sixth leading cause of death from cancer in Japan, with statistics from 2002 indicating a total of about 16 000 deaths from this disease. As a result of the lack of characteristic early symptoms, biliary tract cancers are often diagnosed at an advanced stage, and the prognosis of patients with advanced biliary tract cancer is dismal. Although systemic treatment is used for advanced disease, the impact of existing chemotherapy is virtually negligible. A large number of agents, including 5-fluorouracil (5-FU), mitomycin-C, and cisplatin, have been tested as single agents or in combination therapies without appreciable efficacy (Hejna *et al*, 1998; van Riel *et al*, 1999; Yee *et al*, 2002). Although recent clinical studies have suggested the potential activity of gemcitabine for the treatment of biliary tract cancer, producing response rates of 8 to 36% (Mezger *et al*, 1998; Raderer *et al*, 1999; Gallardo *et al*, 2001; Gebbia *et al*, 2001; Kubicka *et al*, 2001; Penz *et al*, 2001; Tsavaris *et al*, 2004), studies on a larger scale are needed to confirm its efficacy. In any case, to improve the prognosis of patients with biliary tract cancer, a clear need exists for new, effective chemotherapeutic agents.

S-1 is a novel orally administered drug that is a combination of tegafur (FT), 5-chloro-2,4-dihydroxypyridine (CDHP), and oteracil potassium (Oxo) in a 1 : 0.4 : 1 molar concentration ratio (Shirasaka *et al*, 1996a). 5-chloro-2,4-dihydroxypyridine is a competitive inhibitor of dihydropyrimidine dehydrogenase, which is involved in the degradation of 5-FU, and acts to maintain efficacious concentrations of 5-FU in plasma and tumour tissues (Tatsumi *et al*, 1987). Oteracil potassium, a competitive inhibitor of orotate phosphoribosyltransferase, inhibits the phosphorylation of 5-FU in the gastrointestinal tract, reducing the serious gastrointestinal toxicity associated with 5-FU (Shirasaka *et al*, 1993). S-1 therapy in athymic nude rats was associated with the retention of a higher and more prolonged concentration of 5-FU in plasma and tumour tissues, when compared with UFT (Shirasaka *et al*, 1996b). The antitumour effect of S-1 has been already demonstrated in a variety of solid tumours: the response rates for advanced gastric cancer (Sakata *et al*, 1998; Koizumi *et al*, 2000), colorectal cancer (Ohtsu *et al*, 2000), non-small-cell lung cancer (Kawahara *et al*, 2001), and head and neck cancer (Inuyama *et al*, 2001) in the late phase II studies conducted in Japan were 44–49, 35, 22, and 29%, respectively. In addition, a recent early phase II study for advanced pancreatic cancer demonstrated a response rate of 21% in 19 patients (Okada *et al*, 2002). The efficacy of S-1 for the treatment of gastrointestinal cancer has also been reported in European patients: the response rates for advanced gastric cancer (Chollet *et al*, 2003) and colorectal cancer (Van den Brande *et al*, 2003) were 32 and 24%, respectively. However, no previous reports have described the efficacy and safety of S-1 for the treatment of biliary tract cancer. Consequently, the present early phase II study was conducted to evaluate the efficacy and safety of S-1 in patients with advanced biliary tract cancer.

## PATIENTS AND METHODS

### Patients

Patients were required to meet the following eligibility criteria: histologically or cytologically confirmed advanced biliary tract cancer; at least one measurable lesion; no history of prior antitumour treatment except resection; a Karnofsky performance status (KPS) of 80–100 points; age of 20–74 years; an estimated life expectancy of at least 2 months; adequate organ function, defined as a white blood cell count of 4000–12 000 mm^−3^, a platelet count ⩾100 000 mm^−3^, a haemoglobin level ⩾10.0 g/dl, a normal serum creatinine level, a serum total bilirubin level ⩽3 times the upper limit of normal, an aspartate aminotransferase and alanine aminotransferase level ⩽2.5 times the upper limits of normal; and written informed consent. Patients who had obstructive jaundice were considered eligible if their bilirubin level could be reduced to within 3 times the upper limit of normal after biliary drainage. The exclusion criteria were as follows: a history of drug hypersensitivity; severe complications, such as infection, heart disease, and renal disease; symptomatic metastasis of the central nervous system; active concomitant malignancy; marked pleural effusion or ascites; watery diarrhoea; and pregnancy or lactation. This study was approved by the institutional review board at the National Cancer Center and conducted in accordance with the Good Clinical Practice guidelines of Japan.

### Treatments

S-1 was administered orally at a dose of 40 mg m^−2^ twice daily after breakfast and dinner. Three initial doses were established according to the body surface area (BSA) as follows: BSA <1.25 m^2^, 80 mg day^−1^; 1.25 m^2^⩽BSA<1.50 m^2^, 100 mg day^−1^; and 1.50 m^2^⩽BSA, 120 mg day^−1^. S-1 was administered at the respective dose for 28 days, followed by a 14-day rest period; this treatment course was repeated until the occurrence of disease progression, unacceptable toxicities, or the patient's refusal to continue. When a grade 3 or greater haematologic or grade 2 or greater nonhaeamatologic toxicity occurred, the temporary interruption of the S-1 administrations was allowed until the toxicity subsided to grade 1 or less. If the daily dose of S-1 was considered to be intolerable, the retreatment dose was reduced by 20 mg day^−1^ (minimum dose, 80 mg day^−1^). If no toxicity occurred, the rest period shortened to 7 days was allowed. If a rest period of more than 28 days was required because of toxicity, the patient was withdrawn from the study. Patients were not allowed to receive concomitant radiation therapy, chemotherapy, or hormonal therapy during the study. Patients maintained a daily journal to record their intake of S-1 and any signs or symptoms that they experienced. S-1 was provided by Taiho Pharmaceutical Co. Ltd (Tokyo, Japan).

### Response and toxicity evaluation

The response after each course was assessed according to the Japan Society for Cancer Therapy Criteria (Japan Society for Cancer Therapy, 1993), which is similar to the World Health Organization Criteria. Briefly, a complete response (CR) was defined as the disappearance of all clinical evidence of the tumour for a minimum of 4 weeks. A partial response (PR) was defined as a 50% or greater reduction in the sum of the products of two perpendicular diameters of all measurable lesions for a minimum of 4 weeks. No change (NC) was defined as a reduction of less than 50% or a less than 25% increase in the sum of the products of two perpendicular diameters of all lesions for a minimum of 4 weeks. Progressive disease (PD) was defined as an increase of 25% or more in the sum of the products of two perpendicular diameters of all lesions, the appearance of any new lesion, or a deterioration in the clinical status that was consistent with disease progression. Primary bile duct lesions were not considered to be measurable lesions because the dimensions of such lesions are difficult to measure accurately.

The response duration was calculated from the day of the first sign of a response until disease progression; time-to-progression (TTP) was calculated from the date of study entry until documented disease progression; and overall survival time was calculated from the date of study entry to the date of death or the last follow-up. The median probability of the survival period and the median TTP were estimated using the Kaplan–Meier method. Compliance was calculated for all treatment courses using the ratio of the total dose actually administered to the scheduled dose.

Physical examinations, complete blood cell counts, biochemistry tests, and urinalyses were performed at least biweekly. Adverse events were evaluated according to the National Cancer Institute Common Toxicity Criteria, version 2.0. Objective responses and adverse events were confirmed by an external review committee.

Analysis was to be performed when 19 patients were enrolled. In this study, the threshold rate was defined as 5% and the expected rate was set as 15%. If the lower limit of the 90% confidence interval exceeded the 5% threshold (objective response in four or more of the 19 patients), S-1 was judged to be effective and we would proceed to the next large-scale study. If the upper limit of the 90% confidence interval did not exceed the expected rate of 15% (no objective response in the 19 patients), S-1 was judged to be ineffective and the study was to be ended. If response was confirmed in 1–3 of the 19 patients, whether to proceed to the next study or not was judged based on the safety and survival data from the present study.

### Pharmacokinetics

A pharmacokinetic study was performed in the first eight patients enrolled in the study. Blood (5 ml) was collected before and 1, 2, 4, 6, 8, 10, and 12 h after the administration of S-1 on day 1 of the first course. The plasma was then separated by centrifugation and stored at −20°C until analysis. Plasma concentrations of FT were quantified using high-performance liquid chromatography with UV detection, and the concentrations of 5-FU, CDHP, and Oxo were quantified using gas chromatography-negative ion chemical ionisation mass spectrometry, as reported previously (Matsushima *et al*, 1997).

Pharmacokinetic parameters, including the maximum plasma concentration (*C*_max_, ng ml^−1^), time to reach *C*_max_ (*T*_max_, h), area under the concentration *vs* time curve for zero to infinity (AUC_0–∞_, ng h ml^−1^), and the elimination half-life (*T*_1/2_, h) were calculated using a noncompartment model and Win-Nonlin software, Version 3.1 (Pharsight, Apex, NC, USA).

## RESULTS

### Patients

Nineteen consecutive eligible patients with advanced biliary tract cancer were enrolled in the study between July 2000 and January 2002 at the National Cancer Center Hospital, Tokyo, Japan. The patient characteristics are summarised in Table 1

**Table 1:** Patient characteristics (*n*=19)

**Characteristics**		**No. of patients (%)**
*Gender*		
Male		12 (63.2)
Female		7 (36.8)
Median age (years) (range)	59 (44–71)	
		
*Karnofsky performance status, points*		
100		8 (42.1)
90		10 (52.6)
80		1 (5.3)
Median body surface area (m^2^) (range)	1.56 (1.37–1.83)	
Median first dose (mg m^−2^ day^−1^) (range)	72.9 (65.8–78.6)	
History of surgical resection		6 (31.6)
		
*Primary tumour site*		
Gallbladder		16 (84.2)
Extrahepatic bile ducts		2 (10.5)
Ampulla of Vater		1 (5.3)
Median CEA (ng ml^−1^) (range)	6.8 (1–737)	
Median CA 19-9 (U ml^−1^) (range)	103 (1–48,160)	

. Before the start of the study, six patients had received surgical resection and seven patients had undergone percutaneous or endoscopic biliary drainage for obstructive jaundice. Of the 19 patients, 17 had metastatic disease at the time of their enrollment in the study, while two patients were diagnosed as having locally advanced disease. The liver was the most common site of metastases (14 patients), followed by the distant lymph nodes (11 patients) and the lungs (three patients).

### Treatments

In all, 19 patients were given a total of 63 courses of chemotherapy, with a median of two courses each (range, 1–12). The initial administered dose of S-1 was 100 mg day^−1^ in seven patients and 120 mg day^−1^ in 12 patients. Dose reduction was required in one patient because of grade 2 diarrhoea after the third course of treatment. The reasons for treatment discontinuation were as follows: disease progression (16 patients), grade 3 diarrhoea and grade 3 stomatitis (one patient), prolonged grade 2 nausea (one patient), and patient's request for transference to another hospital (one patient). Except for two patients, in whom treatment was abandoned because of toxicities, all the patients were treated as outpatients. The overall compliance rate was 94.3%.

### Response and survival

Of the 19 patients, none of the patients showed a CR but four patients achieved a PR, giving an overall response rate of 21.1% (95% confidence interval, 6.1–45.6%) (Table 2

**Table 2:** Response results (*n*=19)

	**Total**	**CR**	**PR**	**NC**	**PD**	**NE**	**Response rate (%)**
Overall	19	0	4	9	5	1	21.1
							
*Primary tumour site*							
Gallbladder	16	0	3	8	4	1	18.8
Extrahepatic bile ducts	2	0	0	1	1	0	0
Ampulla of Vater	1	0	1	0	0	0	100.0

**Figure 1:**
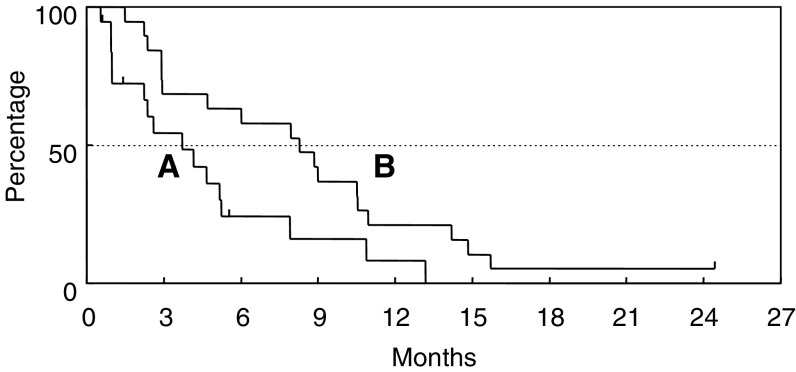
Time to progression (**A**) and overall survival time (**B**).

CR=Complete response; PR=partial response; NC=no change; PD=progressive disease; NE=not evaluable.

). The median response duration was 6.7 months (range, 2.8–10.0 months). Nine patients showed NC and five patients had PD. The tumour response could not be evaluated in one patient because the patient was transferred to another hospital, for personal reasons, prior to the response evaluation. At the time of analysis, 18 of the 19 patients had died because of disease progression. The median TTP was 3.7 months, and the overall median survival time was 8.3 months, with a 1-year survival rate of 21.1% (Figure 1

).

### Toxicity

All 19 patients were assessed for toxicities that are listed in Table 3

**Table 3:** Treatment-related adverse events (*n*=19): worst grade reported during treatment period

	**Grade^a^**					
**Toxicity**	**1**	**2**	**3**	**4**	**Grade 1–4 (%)**	**Grade 3–4 (%)**
*Haematologic*						
Leukopenia	5	3	0	0	42.1	0
Neutropenia	4	2	1	0	36.8	5.3
Anaemia	3	4	1	0	42.1	5.3
Thrombocytopenia	2	0	0	0	10.5	0
						
*Nonhaematologic*						
Nausea	4	2	1	0	36.8	5.3
Vomiting	4	0	0	0	21.1	0
Anorexia	3	0	2	0	26.3	10.5
Stomatitis	3	0	1	0	21.1	5.3
Diarrhoea	2	2	1	0	26.3	5.3
Total bilirubin	1	1	0	0	10.5	0
ALT	2	4	0	0	31.6	0
AST	4	2	0	0	31.6	0
Fatigue	0	0	2	0	10.5	10.5
Fever	0	0	1	0	5.3	5.3
Rash	1	0	0	0	5.3	0
Pigmentation changes	3	0	0	0	15.8	0

AST=aspartate aminotransferase; ALT=alanine aminotransferase.

a NCI Common Toxicity Criteria, version 2.0.

. Treatment was generally well tolerated throughout the study. Although haematologic and gastrointestinal toxicities were common, most of the toxicities were mild and transient. Grade 3 anorexia and fatigue occurred in two patients each (10.5%), and grade 3 anaemia, neutropenia, stomatitis, nausea, diarrhoea, and fever occurred in one patient each (5.3%). No signs of cumulative toxicity were noted. Of the 17 patients who were treated as outpatients, one patient required hospitalisation because of grade 3 nausea, anorexia, and fatigue during the middle of the first course of treatment. Although one patient died within 8 weeks of study enrollment because of rapid disease progression, no treatment-related deaths were observed.

### Pharmacokinetics

Table 4

**Table 4:** Pharmacokinetic parameters after single administration of S-1 at a dose of 40 mg m^−2^

**Compound**	**Parameter**	**Current study (*n*=8)**	**Hirata's study (*n*=12)**
FT	*C*_max_ (ng ml^−1^)	1721.6±400.4	1971.0±269.0
	*T*_max_ (h)	3.6±1.1	2.4±1.2
	AUC (ng h ml^−1^)	24643.0±7915.0^a^	28216.9±7771.4^b^
	*T*_1/2_ (h)	8.2±2.0	13.1±3.1
			
5-FU	*C*_max_ (ng ml^−1^)	146.9±62.1	128.5±41.5
	*T*_max_ (h)	4.0±0.0	3.5±1.7
	AUC (ng h ml^−1^)	799.8±285.3^a^	723.9±272.7^c^
	*T*_1/2_ (h)	1.9±0.3	1.9±0.4
			
CDHP	*C*_max_ (ng ml^−1^)	245.3±64.9	284.6±116.6
	*T*_max_ (h)	3.3±1.0	2.1±1.2
	AUC (ng h ml^−1^)	1472.6±381.6^a^	1372.2±573.7^b^
	*T*_1/2_ (h)	3.2±0.7	3.0±0.5
			
Oxo	*C*_max_ (ng ml^−1^)	55.3±48.4	78.0±58.2
	*T*_max_ (h)	3.3±1.0	2.3±1.1
	AUC (ng h ml^−1^)	230.6±140.2^a^	365.7±248.6^d^
	*T*_1/2_ (h)	2.8±0.6	3.0±1.4

Parameters are represented as mean±s.d.

a AUC_0–∞_.

b AUC_0–48_.

c AUC_0–14_.

d AUC_0–24_.

FT=tegafur; 5-FU=5-fluorouracil; CDHP=5-chloro-2,4-dihydroxypyridine; Oxo=oteracil potassium.

and Figure 2

**Figure 2:**
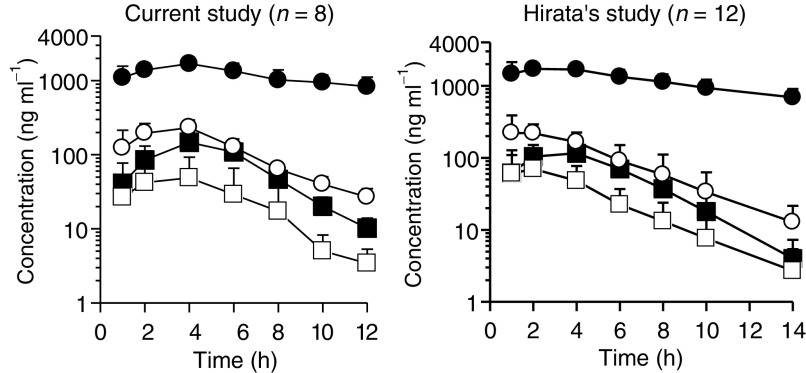
Plasma concentration–time profiles of FT (•), 5-FU (▪), CDHP (○), and Oxo (□) after administration of S-1. The values are expressed as the mean±s.d.

show the results of the pharmacokinetic study for S-1 in the current study. The pharmacokinetic parameters for S-1 in other cancers, as reported by Hirata *et al* (1999) are also shown in Table 4 and Figure 2 for reference. Hirata *et al* investigated the pharmacokinetic parameters after the single administration of S-1 at a dose of 40 mg m^−2^ in 12 Japanese patients with gastric, colorectal, and breast cancer. The parameters of 5-FU in both studies were similar, and no large differences in the parameters of other compounds, including CDHP, were seen.

## DISCUSSION

Although most patients with biliary tract cancer have an unresectable disease at the time of diagnosis, no standard chemotherapies have been established for this disease (Hejna *et al*, 1998; van Riel *et al*, 1999; Okusaka, 2002; Yee *et al*, 2002). Since biliary tract cancer is an uncommon disease, studies of chemotherapy for biliary tract cancer are relatively few, and the number of included patients is generally small. In addition, the response rates and survival times described in published studies are difficult to compare because most studies contain patients with heterogeneous tumour groups, such as intrahepatic or extrahepatic bile duct cancer and gallbladder cancer. 5-fluorouracil has been the most commonly studied drug for this disease, although the antitumour effect of single-agent 5-FU is limited, with a response rate of less than 20%. Although the combined use of 5-FU with other agents, such as leucovorin, mitomycin C, or cisplatin, often produces a response rate of over 20% (Polyzos *et al*, 1996; Ducreux *et al*, 1998; Taïeb *et al*, 2002), the toxicities also become greater; whether combination therapies contribute to prolonged survival remains uncertain. In recent small-scale studies, gemcitabine has shown relatively good response rates, ranging from 8 to 36%, for biliary tract cancer (Mezger *et al*, 1998; Raderer *et al*, 1999; Gallardo *et al*, 2001; Gebbia *et al*, 2001; Kubicka *et al*, 2001; Penz *et al*, 2001; Tsavaris *et al*, 2004), but large-scale studies are needed to confirm its efficacy. Therefore, the development of new effective chemotherapeutic agents is urgently needed to improve survival in patients with advanced biliary tract cancers.

A novel orally administered drug, S-1, has been developed based on the biochemical modulations by CDHP, a dihydropyrimidine dehydrogenase inhibitor, and Oxo, a protector against 5-FU-induced gastrointestinal toxicity; S-1 has exhibited significant antitumour effects on various solid cancers (Sakata *et al*, 1998; Koizumi *et al*, 2000; Ohtsu *et al*, 2000; Inuyama *et al*, 2001; Kawahara *et al*, 2001; Chollet *et al*, 2003; Van den Brande *et al*, 2003). Since the drug is available in oral form, S-1 has a potential advantage, as far as patient convenience is concerned, especially in terms of quality-of-life. This consideration is very important for biliary tract cancer patients because their remaining lifespan is generally short. Consequently, the efficacy of S-1 for the treatment of biliary tract cancer was examined.

In the current study, S-1 produced a good response rate of 21.1%, which is superior to those obtained with other single agents, including 5-FU, mitomycin C, and cisplatin (Table 5

**Table 5:** Recent studies of single-agent chemotherapy for biliary tract cancer

		**No. of patients**			
**Author**	**Regimen**	**Total**	**Gallbladder Ca.**	**Response rate (%)**	**MST (months)**
Takada *et al* (1994)	5-FU	18	10	0	NA
Taal *et al* (1993)	Mitomycin C	30	13	10	4.5
Okada *et al* (1994)	Cisplatin	13	6	8	5.5
Jones *et al* (1996)	Paclitaxel	15	4	0	NA
Pazdur *et al* (1999)	Docetaxel	17	0	0	NA
Papakostas *et al* (2001)	Docetaxel	25	16	20	8
Sanz-Altamira *et al* (2001)	Irinotecan	25	10	8	10
Mezger *et al* (1998)	Gemcitabine	13	4	8	NA
Raderer *et al* (1999)	Gemcitabine	19	5	16	6.5
Penz *et al* (2001)	Gemcitabine^a^	32	10	22	11.5
Kubicka *et al* (2001)	Gemcitabine	23	0	30	9.3
Gallardo *et al* (2001)	Gemcitabine	26	26	36	7
Gebbia *et al* (2001)	Gemcitabine	18	12	22	8
Tsavaris *et al* (2004)	Gemcitabine	30	14	30	14
Current study	S-1	19	16	21	8.3

5-FU: 5-fluorouracil; MST: median survival time; NA: not available.

a Biweekly.

), suggesting an antitumour effect of S-1 on biliary tract cancer. In this study, patients with gallbladder cancer accounted for three of the four responders; however, the efficacy of S-1 for each primary tumour site cannot be accurately assessed because of the small number of subjects analysed.

Since patients with biliary tract cancer tend to suffer various tumour-related complications, such as cholangitis and impaired liver function, enhanced chemotherapy-related toxicities, including neutropenic sepsis, are a concern. However, S-1 was well tolerated in the present study, and no grade 4 toxicities occurred. Haematological toxicities were acceptable and similar to the results of clinical studies examining S-1 for the treatment of other cancers in Japan. Gastrointestinal toxicities were also well tolerated, as in the other Japanese studies, although strong gastrointestinal toxicities, particularly severe diarrhoea, have been reported in Western countries (van Groeningen *et al*, 2000; Cohen *et al*, 2002; Chollet *et al*, 2003; Van den Brande *et al*, 2003). The difference in toxicities between the Japanese and Western studies remains unexplained, although the conversion of FT to 5-FU seems to occur more slowly in Japanese patients than in patients from other ethnic groups (Comets *et al*, 2003). A pharmacokinetic study suggested that the pharmacokinetic parameters of S-1 were similar in patients with biliary tract cancer and in patients with other cancers in Japan.

Since no serious adverse events occurred in this study, most of the patients were treated as outpatients, enabling a relatively good quality-of-life. The S-1 compliance rate of the patients was very good (94.3%), with only one patient requiring a dose reduction and only two patients discontinuing S-1 because of toxicity. In view of the favourable toxicity profile, its evaluation in combination with other agents might be of particular interest to improve therapeutic results. Combination therapy with S-1 and cisplatin has already been conducted for gastric cancer, and an excellent response rate of 76% was reported in a phase II study (Ohtsu *et al*, 2001).

In conclusion, the results of this study indicate that S-1 is a safe and active agent for the treatment of patients with biliary tract cancer. Further investigations of this agent are warranted in this population of patients with a poor prognosis.
